# Human Protein Phosphatase PP6 Regulatory Subunits Provide Sit4-Dependent and Rapamycin–Sensitive Sap Function in *Saccharomyces cerevisiae*

**DOI:** 10.1371/journal.pone.0006331

**Published:** 2009-07-21

**Authors:** Helena Morales-Johansson, Rekha Puria, David L. Brautigan, Maria E. Cardenas

**Affiliations:** 1 Department of Molecular Genetics and Microbiology, Duke University Medical Center, Durham, North Carolina, United States of America; 2 Center of Cell Signaling, University of Virginia School of Medicine, Charlottesville, Virginia, United States of America; Research Institute for Children and the Louisiana State University Health Sciences Center, United States of America

## Abstract

In the budding yeast *Saccharomyces cerevisiae* the protein phosphatase Sit4 and four associated proteins (Sap4, Sap155, Sap185, and Sap190) mediate G_1_ to S cell cycle progression and a number of signaling events controlled by the target of rapamycin TOR signaling cascade. Sit4 and the Sap proteins are ubiquitously conserved and their human orthologs, PP6 and three PP6R proteins, share significant sequence identity with their yeast counterparts. However, relatively little is known about the functions of the PP6 and PP6R proteins in mammalian cells. Here we demonstrate that the human PP6R proteins physically interact with Sit4 when expressed in yeast cells. Remarkably, expression of PP6R2 and PP6R3 but not expression of PP6R1 rescues the growth defect and rapamycin hypersensitivity of yeast cells lacking all four Saps, and these effects require Sit4. Moreover, PP6R2 and PP6R3 enhance cyclin G_1_ gene expression and DNA synthesis, and partially abrogate the G_1_ cell cycle delay and the budding defect of the yeast quadruple *sap* mutant strain. In contrast, the human PP6R proteins only modestly support nitrogen catabolite gene expression and are unable to restore normal levels of eIF2α phosphorylation in the quadruple *sap* mutant strain. These results illustrate that the human PP6-associated proteins are capable of providing distinct rapamycin-sensitive and Sit4-dependent Sap functions in the heterologous context of the yeast cell. We hypothesize that the human Saps may play analogous roles in mTORC1-PP6 signaling events in metazoans.

## Introduction

Protein dephosphorylation is an essential enzymatic activity through which cell signaling in response to diverse cues is processed and cellular events evoked by protein phosphorylation are mechanistically reversed. In the yeast *Saccharomyces cerevisiae* the type 2A-like protein phosphatase catalytic subunit Sit4 functions downstream of the rapamycin-sensitive TOR complex 1 (TORC1) to govern responses to nutrients and events required for normal G_1_ to S phase cell cycle transition and budding [1], [2]. In response to nutrient signals TORC1 modulates interaction of Sit4 with its essential effector Tap42 and thereby regulates translation, expression of the nitrogen catabolite-repressed (NCR) and retrograde response genes, and the Pkc1 cell integrity pathway [3]–[6].

Homologs of Sit4 and Tap42, known as PP6 and alpha4, respectively have been identified in mammalian cells. Moreover, pairwise interactions between PP2A, PP6, and alpha4 have been characterized [7]. Although rapamycin-sensitive functions have been ascribed to the PP2A-alpha4 protein phosphatase, a role for the PP6-alpha4 holoenzyme in mTORC1signaling has not yet been demonstrated [8]–[10]. PP6 shares 61% amino acid sequence identity with Sit4 and functionally complements *sit4* mutations in *S. cerevisiae* and of the Sit4 homolog ppe1 in *Schizosaccharomyces pombe* [11].

In addition, Sit4 also interacts with four proteins known as the Saps (Sit4 associated proteins). Because either deletion of *SIT4* or all four *SAP* genes results in similar phenotypic consequences, including delayed G_1_ to S phase cell cycle progression and thereby slower growth, budding defects, and impaired NCR gene expression and Gcn2-regulated translation, it has been thought that Sit4 functions in concert with the Saps to control these cellular processes [2], [3], [5], [12]–[14]. Based on amino acid sequence identity and functional analysis, the four Saps can be classified into two distinct subgroups: Sap185 and Sap190 are more similar to each other than to Sap155 and Sap4 [12]. Also Sap185 and Sap190 share similar functions not shared with Sap155 and Sap4. Thus, Sap185 and Sap190 function together with Sit4 to provide an essential role in the absence of Bem2 (most likely in regulating the Pkc1-cell integrity pathway), and are required for eIF2α dephosphorylation and to render yeast cells sensitive to the toxin zymocin (produced by *Kluyveromyces lactis*) [5], [6], [12], [15]. Moreover, Sap155 and Sap185 perform distinct, albeit in this case opposite, functions in K^+^ efflux regulation [16]. Finally, Sap185, Sap190, or Sap155 are individually equally effective in mediating NCR gene expression [5]. Thus, based on these criteria Sap4 is less functionally effective than the other three Saps and, similar to cells lacking the four Saps, cells expressing only Sap4 (*sap155 sap185 sap190* triple mutant cells) grow poorly, show defects in NCR gene expression, and are hypersensitive to rapamycin and resistant to zymocin [1], [5], [15]. These and other genetic data are consistent with a model in which the Sap proteins act positively and in combination with Sit4 to regulate phosphatase activity, substrate specificity, or both [5], [6], [12], [15].

Recently, combined sequence and biochemical analysis uncovered Sap homologs in diverse organisms including other fungi, amphibians, worms, plants, flies, and metazoans, underscoring a potentially widespread biological conservation of Sap function [17]. Interestingly, the three human Saps identified, named PP6R1, PP6R2, and PP6R3, were shown to physically interact with human PP6 and share limited amino acid sequence identity (10%) with the four yeast Saps [17]. PP6R1 and PP6R3 share nearly identical tissue distribution, and are enriched in lung, spleen, and the bladder whereas PP6R2 is mainly present in bladder. Subsequently, it was shown that PP6, together with a PP6R and an Ankyrin repeat subunit, forms holoenzyme trimers that effectively interact with ΙκΒε in response to TNFα [17], [18]. These results are consistent with a role for PP6-PP6R complexes in limiting NF-κβ signaling; however, little else is known about PP6Rs function and any involvement in mTor signaling has not as yet been demonstrated.

In this study we have examined if the human PP6R proteins are functional Sap homologs by testing their ability to provide Sap function in *S. cerevisiae*. We find that human PP6R2, PP6R3, and to a lesser extent PP6R1, are stably expressed and physically interact with Sit4 in yeast cells. Expression of the individual PP6R2 and PP6R3 proteins rescued the growth defect and rapamycin hypersensitivity of yeast cells lacking all four Saps in a Sit4-dependent fashion. Sit4 and the Sap proteins function in G_1_ to promote timely DNA replication and bud formation [2], [12]. Both PP6R2 and PP6R3 but not PP6R1 partially provided these G_1_ functions when expressed in the quadruple *sap* mutant strain. In addition, the human PP6R proteins have a modest effect in restoring NCR gene expression in response to rapamycin treatment in cells devoid of the Saps. In contrast, none of the human PP6Rs were capable of restoring normal eIF2α phosphorylation levels in either a quadruple *sap* mutant or a *sap185 sap190* double mutant strain. PP6R2 and PP6R3 partially restored zymocin sensitivity to cells lacking Sap185 and Sap190. Taken together, these results illustrate that the PP6-associated proteins are capable of providing distinct rapamycin-sensitive, Sit4-dependent Sap functions in yeast cells. By extension, we hypothesize that the human Saps may play related roles in TORC1-PP6 signaling events in metazoans.

## Results

### The human PP6R proteins are stably expressed in yeast cells and physically interact with Sit4

**Figure 1:**
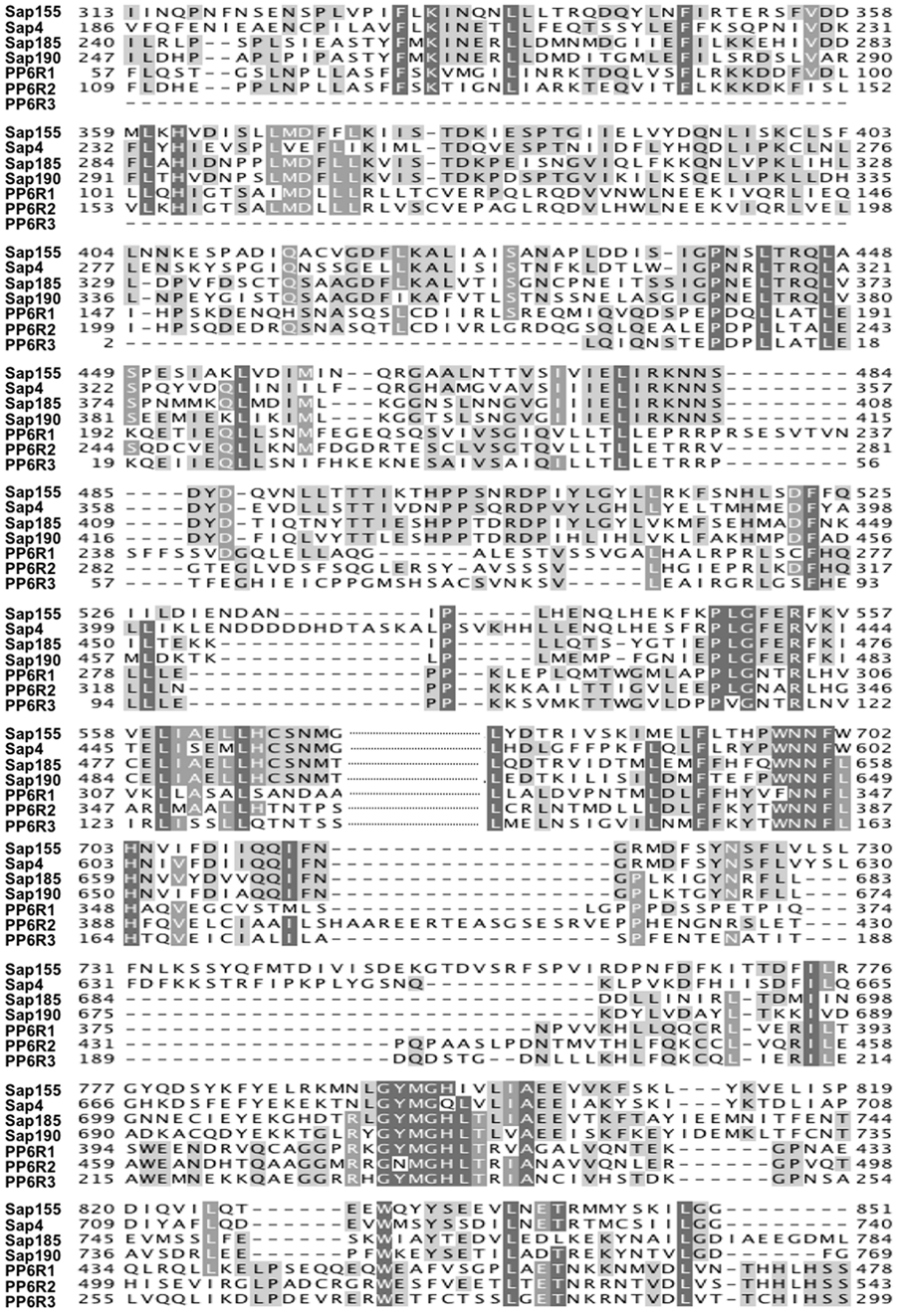
The human PP6R and the yeast Saps proteins share marked homology within the SAPS domain. The protein sequences of the SAPS domain of *Saccharomyces cerevisiae* Saps and *Homo sapiens* PP6Rs were aligned using Jalview. Amino acid conservation is highlighted with grey boxes where the darkest grey indicates the highest level of similarity. Dashed line denotes non-homologous region.

**Figure 2:**
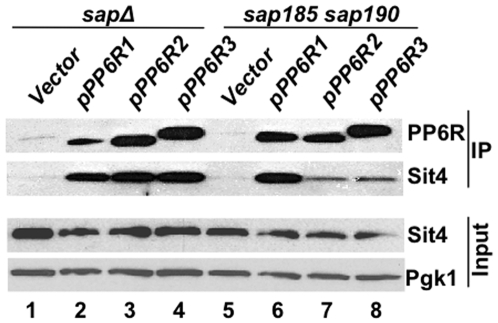
PP6R proteins associate with the PP2A-like phosphatase catalytic subunit Sit4. The quadruple *sap* mutant (JRY40) and *sap185 sap190* double mutant (JRY29) strains were transformed with vector and derivatives expressing N-terminal FLAG tagged versions of *PP6R1*, *PP6R2* and *PP6R3*. Proteins were immunoprecipitated with anti-FLAG antibody and analyzed by western blotting. The membrane was stripped and interaction with Sit4 was detected by reprobing with a Sit4 specific antiserum. Sit4 was also detected in equivalent amounts of protein extracts and Pgk1 served as a loading control.

PP6 and Sit4 share a high degree of amino acid sequence identity; however, the PP6R proteins exhibit limited identity with the yeast Saps (Figure 1). This homology is largely confined to the SAPS domain (as defined by the Protein families database of alignments - Pfam), a central four hundred amino acid region containing several discrete, common motifs with the ability to form α helices [17]. The striking conservation between the yeast Saps and the PP6R proteins prompted us to explore the functions of the PP6R proteins in yeast cells. To this end, FLAG epitope-tagged versions of PP6R1, PP6R2, and PP6R3 were PCR amplified from previously published pcDNA3-FLAG constructs [17], placed under the control of the *ADH1* constitutive promoter in the centromeric pRS416-*ADH* vector, and expressed in yeast cells lacking the four *SAP* genes. All three PP6R proteins were effectively expressed as detected by immunoprecipitation and western blot analysis; however, PP6R2 and PP6R3 were expressed at a higher level than PP6R1 (compare lanes 2–4 in Figure 2).

Because all characterized Sap functions are known to require Sit4, it was therefore important to examine if the PP6R proteins interact with Sit4. As shown in Figure 2 (lanes 2–4), PP6R1, PP6R2, and PP6R3 effectively co-immunoprecipitated with Sit4 when expressed in the quadruple *sap* mutant. Interestingly, whereas the PP6R1-Sit4 interaction was robust in the *sap185 sap190* double mutant, which still expresses Sap155 and Sap4, the PP6R2 and PP6R3-Sit4 interactions in this strain were reproducibly weaker than those observed in the quadruple *sap* mutant (Figure 2, lanes 6–8). These results suggest that PP6R1 competes more effectively than PP6R2 and PP6R3 with Sap155 for Sit4 interaction. This is in accord with previous observations that the Sap proteins compete with each other for Sit4 binding and that Sap155 outcompetes the other Saps in this function [12], [19] (and see further evidence below).

### Human PP6R proteins support growth and abrogate rapamycin hypersensitivity of Sap-defective yeast mutants

**Figure 3:**
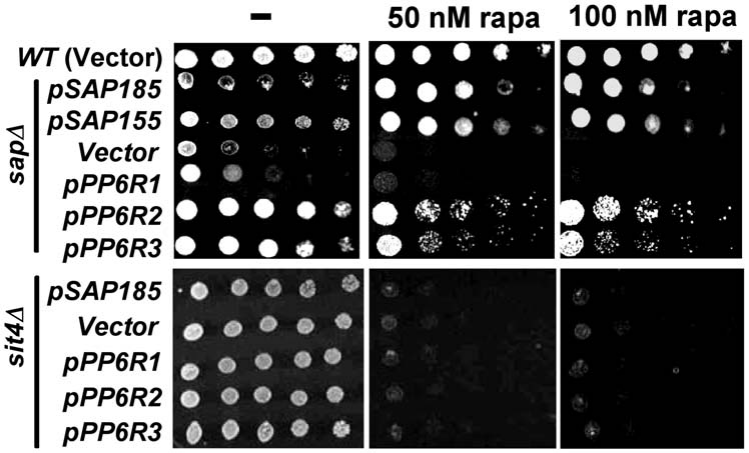
PP6R2 and PP6R3 rescue the growth defect and rapamycin hypersensitivity of yeast cells lacking all *SAP*s. Isogenic WT (MLY41) strain transformed with vector, quadruple *sap* (JRY40), *sit4* (SCY94) and *sap185 sap190* (JRY29) mutant strains transformed with vector and plasmids expressing *SAP185*, *SAP155*, *PP6R1*, *PP6R2*, and *PP6R3* were grown overnight in SD-Ura medium. Equivalent numbers of cells were serially diluted, and aliquots were spotted onto SD-Ura plates containing drug vehicle or 50 nM and 100 nM rapamycin. After 3 days of incubation at 30°C, the plates were photographed.

*S. cerevisiae* mutant cells lacking the four *SAP* genes grow poorly and are hypersensitive to the Tor inhibitor rapamycin. To investigate if the PP6R proteins can provide Sap function in yeast, we tested their ability to suppress these mutant phenotypes. Strikingly, similar to expression of Sap185 and Sap155, expression of PP6R2 and PP6R3 but not PP6R1 greatly improved the growth of the quadruple *sap* mutant strain and moreover, restored the rapamycin sensitivity of these cells to a level comparable to that observed in the WT strain (Figure 3). In contrast, expression of either Sap185 or the PP6R proteins in a *sit4* deleted strain, which also exhibits severe growth defect and rapamycin hypersensitivity, failed to mitigate either phenotype. These results demonstrate that the PP6R effects on cell growth and rapamycin sensitivity are Sit4-dependent (Figure 3). Intriguingly, the expression of PP6Rs failed to alleviate the rapamycin hypersensitivity characteristic of *sap185 sap190* double mutant cells (Figure 3). This result correlates well with the observation that PP6R2 and PP6R3 are not effective in competing with Sap155 and Sap4 for Sit4 binding and suggest that, despite the robust PP6R1-Sit4 interaction, PP6R1 is unable to provide Sap function (Figure 2). These data illustrate that the PP6R2 and PP6R3 proteins are capable of supporting Sap-Sit4-dependent growth and can provide rapamycin-sensitive function in a Sit4-dependent fashion in yeast cells.

### PP6R2 and PP6R3 can partially promote G_1_ progression through DNA replication and bud formation

**Figure 4:**
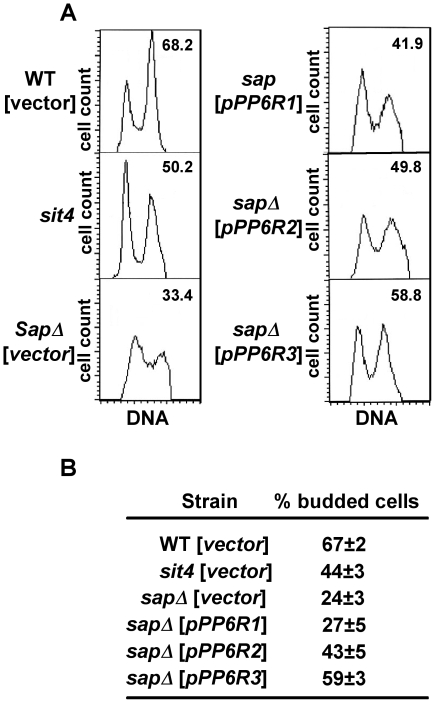
PP6R proteins partially overcome the G_1_ delay and budding defects of quadruple *sap* mutant cells. Isogenic WT (MLY41) and *sit4* (SCY94) strains transformed with vector, and quadruple *sap* (JRY40) mutant strain transformed with vector and plasmids expressing *PP6R1*, *PP6R2* and *PP6R3* were grown to exponential phase and prepared for flow cytometry analysis. The plots show cell count (vertical axis) versus fluorescence intensity, which is proportional to DNA content (horizontal axis). The percentage of cells with 2N DNA content is shown in the upper right corner of each panel. (B) Table shows the percentage of budded cells, which was determined in cell cultures used for the flow cytometry. Results shown are representative of three independent experiments.

Sit4 is required for cell cycle transition from late G_1_ phase to S phase [2], [20]. Deletion of the *SIT4* gene or deletion of the four *SAP* genes results in slow growth with a large population of unbudded cells and an increase in the number of cells with a 1N DNA content, characteristic of a G_1_ cell cycle arrest [12], [20]. This prompted us to examine whether the PP6R proteins act by reversing the G_1_ cell cycle arrest of the quadruple *sap* mutant. We note that asynchronous cells from the quadruple *sap* mutant of the ∑1278b background have a tendency to aggregate, complicating cell sorting by FACS analysis. However, despite this limitation we reproducibly observed an increase in the number of cells containing 2N DNA (1.5- and 1.8-fold, respectively) when PP6R2 and PP6R3, but not PP6R1, were expressed in the quadruple *sap* mutant (Figure 4). This result is in agreement with the observed failure of PP6R1 to overcome the growth defect of cells lacking the four Saps (Figure 2).

The increased 1N DNA content of the *sit4* and the quadruple *sap* mutant strains is associated with a large fraction of unbudded cells in exponentially growing cultures [12], [20]. To investigate if the PP6R proteins promote budding, we monitored the percentage of budded cells in exponentially growing cultures of the quadruple *sap* mutant strain expressing these proteins. Unlike PP6R1, expression of PP6R2 and PP6R3 resulted in an increase (1.8- and 2.5-fold, respectively) in the fraction of budded cells (Figure 4B).

**Figure 5:**
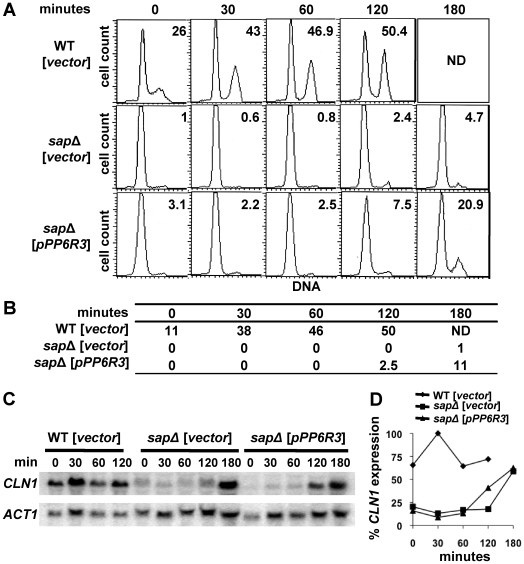
PP6R3 partially enhances *CLN1* expression and cell cycle progression. Isogenic WT (MLY41) and quadruple *sap* (JRY40) mutant strains transformed with vector or plasmid expressing *PP6R3* were grown to exponential phase and elutriated to enrich for G_1_ cells. Elutriated cells were collected, transferred to fresh SD-Ura media, and samples were taken over a 3 hours period for G_1_ functions analysis. (A) Samples were analyzed for DNA content by flow cytometry as in Figure 4A. The percentage of cells with 2N DNA content is shown in the upper right corner of each panel). (B) Table shows the percentage of budded cells (ND, not detectable). (C) *CLN1* and *ACT1* mRNA expression was assessed by northern blot. (D) Northern blot signals from experiment shown in Panel C for *CLN1* were quantified and normalized to the *ACT1* loading control signal. Results shown are the relative percentage of gene expression with maximal level of expression at 30 min for WT as 100%.

The Sit4 and Sap proteins are also required for normal expression of G_1_ cyclins [2], [12]. To further study the effect of PP6R3 expression on Sit4 functions in cell cycle progression, we monitored bud emergence and the induction of G_1_ cyclins in synchronized cultures. To this end, G_1_-phase daughter cells from the wild-type and quadruple *sap* mutant were collected by centrifugal elutriation and cells were allowed to progress through the cell cycle. *Saccharomyces cerevisiae* cells are best sorted by centrifugal elutriation when grown in media containing sucrose as the sole carbon source (D.J. Lew, personal communication). However, we found that the quadruple *sap* mutant cells grow very poorly in sucrose media. To obviate this limitation we grew the cells in dextrose containing media. Under these growth conditions, elutriation of the WT strain yielded an enrichment of 74% of cells with a 1N DNA content, whereas elutriation of the quadruple *sap* mutant mostly resulted in cells with a 1N DNA content (see panels for zero time points in Figure 5A). Upon incubation of elutriated G_1_-cells in fresh media an increase in *CLN1* expression was observed when wild-type cells resumed growth as expected (Figure 5C and 5D). Interestingly, whereas cells transformed with the vector alone showed *CLN1* expression at the 3 hr time point, expression of PP6R3 resulted in an earlier and increased expression of *CLN1* at the 2 hr time point (Figure 5C and 5D). These results correlated with a four-fold increase in the percentage of PP6R3 expressing-cells with a 2N DNA content (∼21%) compared to the quadruple *sap* mutant cells transformed with vector alone (4.7%) (Figure 5A). In accord with this result, we also observed an increase in the budding index of the quadruple *sap* mutant cells expressing PP6R3 (11%) versus cells transformed with vector (1%) (Figure 5B). Taken together, these data show that PP6R2 and PP6R3 are able to promote G_1_ progression in yeast cells whereas PP6R1 is not.

### The human PP6R proteins support a modest level of NCR gene expression but fail to promote eIF2α dephosphorylation

**Figure 6:**
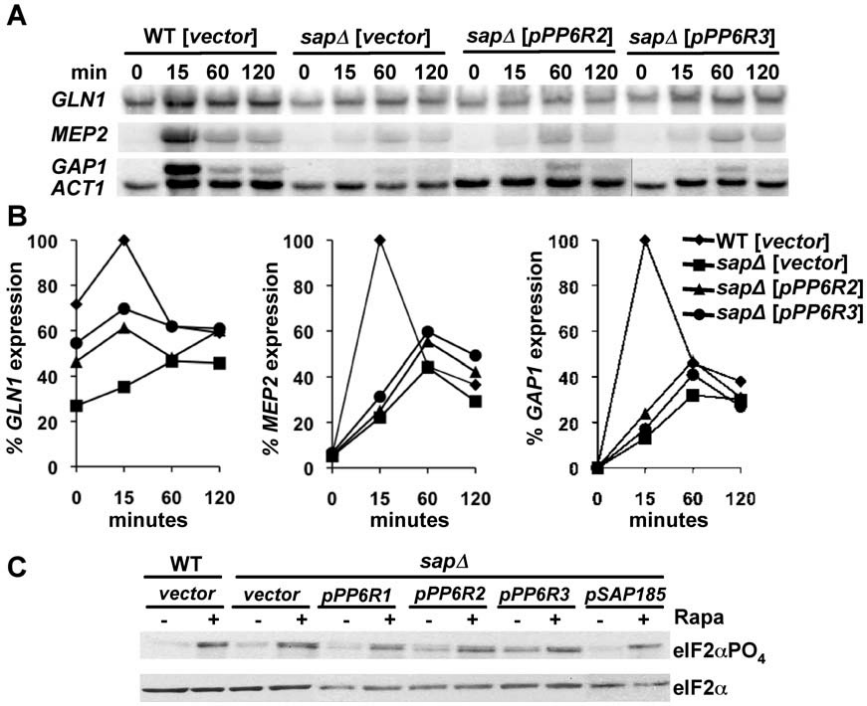
PP6R2 and PP6R3 fail to restore normal levels of NCR response in cells lacking all *SAP* genes. (A) Exponentially growing cultures of the quadruple *sap* mutant strain (JRY40) transformed with vector or its derivatives expressing *PP6R2* and *PP6R3* were treated with rapamycin over a 2 hr period. RNA was isolated at the indicated time points *GLN1*, *MEP2*, *GAP1*and *ACT1* mRNA expression levels were assayed by northern blot. (B) Northern blot signals from Panel A were quantified and normalized to the *ACT1* loading control signal. Results shown are the relative percentage of gene expression with maximal level of expression at 15 min as 100%. (C) PP6R proteins fail to restore normal levels of eIF2α dephosphorylation to the quadruple *sap* mutant strain (JRY40). Exponentially growing cultures of isogenic WT (MLY41) strain transformed with vector, and the quadruple *sap* mutant (JRY40) strain transformed with vector and derivatives expressing *PP6R1*, *PP6R2*, *PP6R3* and *SAP185* were treated with 100 nM rapamycin for 0 or 20 min. Whole-cell extracts were prepared and analyzed by sequencial western blotting with antibodies specific for the phosphorylated and non-phosphorylated form of eIF2α.

We have previously shown that expression of the NCR genes in response to either rapamycin treatment or ammonium limitation requires Sit4 and one of the Sap proteins. Sap155, Sap185, or Sap190 alone are equally effective; however, Sap4 is unable to support this function [5]. The yeast *sap* quadruple mutant individually expressing PP6R2 and PP6R3 was treated with rapamycin for various periods of time. In agreement with previous results, in wild type cells (transformed with the vector control) rapamycin triggered the expression of the NCR genes *GLN1*, *MEP2*, and *GAP1* and rapamycin treatment largely failed to activate these genes in the quadruple *sap* yeast mutant transformed with the vector alone (Figure 6A and 6B). Expression of PP6R2 and PP6R3 resulted in a modest and delayed NCR gene expression (Figure 6A and 6B), indicating that despite effective interaction with Sit4 these human Sap proteins are only modestly effective in providing the Sit4-dependent function required for the NCR response.

Exposing yeast cells to amino acid starvation, nutrient limitation, or inhibition of Tor with rapamycin all result in activation of the Gcn2 kinase and initiation of the general amino acid control response [13], [14], [21]. In turn, activated Gcn2 phosphorylates and thereby inactivates the translation initiation factor eIF2α, resulting in a lower level of general translation and preferential translation of a small subset of mRNAs (such as the *GCN4* mRNA), which feature multiple short open reading frames via which translation is regulated [22]. Gcn4 is a master transactivator that promotes expression of ∼400 genes, including those encoding enzymes involved in amino acid biosynthesis [23]. We have characterized a role for Sit4, Sap185, and Sap190 in maintaining a low level of phosphorylated eIF2α to promote general translation [5].

We next examined if the PP6R proteins are able to restore normal levels of eIF2α phosphorylation in quadruple *sap* mutant cells. As expected, eIF2α phosphorylation was increased in wild-type cells treated with rapamycin or in cells lacking the four *SAP* genes (Figure 6C and [5], [13]). Ectopic expression of *SAP185* effectively restored the wild type level of eIF2α phosphorylation in the quadruple *sap* mutant, however expression of the three individual PP6R proteins failed to promote eIF2α dephosphorylation (compare control lanes without rapamycin in Figure 6C).

### PP6R proteins partially mediate *K. lactis* toxin zymocin sensitivity in yeast *sap* mutants

Exposure of *S. cerevisiae* cells to the *K. lactis* toxin zymocin results in a G_1_ cell cycle arrest. The zymocin target is Elongator, an RNA polymerase II-associated histone acetylase multisubunit complex that facilitates PolII-dependent transcription. It has been shown that *sit4* and *sap* mutants lacking Sap185 and Sap190 are resistant to zymocin toxicity [15]. Moreover, Sit4 in combination with Sap185 and Sap190 facilitates zymocin toxicity and acts by dephosphorylating Tot1, which is the largest subunit of the Elongator complex [19]. Zymocin is a trimeric homotoxin composed of α, β, and γ subunits. It has been hypothesized that interaction of the γ subunit with dephosphorylated Tot1 sequesters PolII, precluding transcription of key G_1_-S phase cell cycle regulators [19].

**Figure 7:**
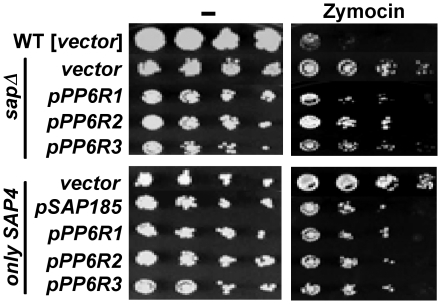
PP6R proteins partially restore zymocin sensitivity to the quadruple *sap* mutant strain. Isogenic WT (MLY41) transformed with vector, the quadruple *sap* mutant (JRY40), and only *SAP4* (*sap155 sap185 sap190*; JRY45) strains transformed with vector or constructs expressing *PP6R1*, *PP6R2*, *PP6R3*, or *SAP185* were grown overnight in SD-Ura medium. Equivalent numbers of cells were serially diluted, and aliquots were spotted onto plates of YPD and YPD containing zymocin. After 3 days of incubation at 30°C, the plates were photographed.

We tested if the mammalian PP6R subunits can substitute for Sap185 and Sap190 to restore zymocin sensitivity to *sap185 sap190* mutants. While the quadruple *sap*, and the *sap155 sap185 sap190* (expressing only *SAP4*) and *sap4 sap155 sap185* (expressing only *SAP190*) triple mutant strains transformed with the vector alone were more resistant to zymocin than the wild type strain, expression of either *SAP185* or any of the three *PP6R* genes restored zymocin toxicity, although not to the same level as that observed in the wild type strain (Figure 7). In this experiment strains were exposed to zymocin in YPD media. We note that although the PP6R proteins are being expressed from an stable maintained, centromeric plasmid, we performed control experiments in which the cells were replica-plated onto plasmid selective medium (SD-Ura) following zymocin exposure to rule out plasmid loss as a trivial explanation for these findings. These results suggest that the PP6R subunits function to mediate interaction with Sit4 targets for zymocin toxicity.

## Discussion

The Saps were originally identified in *S. cerevisiae* as Sit4 associated proteins [12], [20]. The Saps were demonstrated to function positively with Sit4 to promote G_1_ to S phase cell cycle progression and budding [12]. Later, it was shown that Sit4 and the Sap proteins perform a critical role downstream from the TORC1 in controlling the expression of nutrient-regulated genes and in governing Gcn2-dependent translation [5], [13], [14]. Moreover, Sit4 in conjunction with Sap185 and Sap190 is required to sensitize cells to zymocin and to counteract rapamycin toxicity [5], [15]. Sit4 and the Sap proteins have been ubiquitously conserved throughout evolution from yeast to humans. The human Sit4 ortholog PP6 is able to complement the growth defects of *sit4* and *pp1* mutants in budding and fission yeast, respectively [11]. However, studies to examine the role of PP6 have been hampered by the fact that PP6 knock down results in high levels of apoptosis [24]. Nonetheless, indirect evidence has suggested that the N-terminal domain of PP6 has a role in G_1_ cell cycle progression by influencing stabilization of cyclin D protein levels [25]. Apart from a role in limiting NF-κβ signaling, little else is known about the possible cellular functions of the PP6R proteins.

Our studies have shown that the PP6R proteins are expressed in yeast cells and are capable of physically interacting with Sit4 in cells lacking the four Sap proteins. While the observed PP6R interaction with Sit4 is robust in cells devoid of Sap proteins, this interaction is reduced for PP6R2 and PP6R3 in yeast cells expressing the endogenous Sap155 and Sap4, supporting earlier results that the Sap155-Sit4 interaction is quite stable and not subject to competition from the other yeast Saps [12]. Remarkably, PP6R2 and PP6R3 but not PP6R1 complement the growth defect of the quadruple *sap* yeast mutant. The growth improvement afforded by the PP6R2 and PP6R3 proteins in the quadruple *sap* mutant is correlated with a decrease in the fraction of cells with a 1N DNA content and concomitant increase in cells with 2N DNA content, and more efficient budding. Furthermore, PP6R3 expression is able to enhance bud emergence and *CLN1* expression, and increase DNA synthesis upon growth resumption of G_1_-synchronized quadruple *sap* mutant cells. These results suggest that the PP6R proteins could perform an analogous role in combination with PP6 to promote G_1_ to S phase cell cycle progression as recently proposed [25]. The modest ability, or inability, of the PP6Rs to restore TORC1-governed NCR gene expression or normal eIF2α phosphorylation levels respectively, in the quadruple *sap* mutant cells could reflect a reduced (or lack of) ability of these proteins to interact with the effectors for these functions. Thus, the precise mechanisms by which the human PP6Rs abrogate the rapamycin hypersensitivy of the yeast quadruple *sap* mutant do not involve these TORC1 functions. However, rapamycin exposure of cells devoid of Sap function might impose a G_1_ block by more than one mechanism and an attractive model is that the PP6Rs ability to support *CLN1* expression in G_1_ and DNA synthesis promotes G_1_ to S phase cell cycle progression by directly relieving one of these blocks.

Our results show that PP6R1 differs from PP6R2 and PP6R3, and fails to overcome the growth and budding defects and the rapamycin hypersensitivity observed in the quadruple *sap* yeast mutant. Although PP6R1 was expressed at a lower level than PP6R2 and PP6R3 in the quadruple *sap* mutant, it showed a robust interaction with Sit4, and was not out-competed by Sap155 in the *sap185* and *sap190* double mutant (Figure 2). We hypothesize that the unusually stable PP6R1-Sit4 interaction results in an inactive phosphatase and this may be the reason why PP6R1 is the least efficient of the PP6R proteins in providing Sap function when heterologously expressed in yeast. Interestingly, binding of the SV40 small t antigen to the PP2A AC dimer (thought to be tight) in place of the B regulatory subunit was shown to virtually inactivate the phosphatase towards most of its known substrates, and this effect are the basis for the oncogenic effects of small t antigen [26].

The Tor proteins were first identified in yeast via genetic analysis of rapamycin resistant mutants [27] and the mammalian mTor ortholog was later identified by biochemical approaches [28], [29]. These studies provided the foundation to define a key cellular signaling conduit that globally controls cellular physiology and growth in response to nutrients and growth factors [30]. Heterologous expression studies demonstrated that the Tor proteins are not only physically but also functionally conserved [31]. While expression of full-length mTor failed to complement yeast *tor1* or *tor2* mutant cells, expression of Tor1-mTor and Tor2-mTor hybrids supported Tor-dependent, rapamycin-sensitive growth of yeast cells [31]. Thus, both the FKBP12-rapamycin binding (FRB) domain and the kinase domain of mTor are fully functional in the context of the yeast cell [31]. Similarly, expression of human FKBP12 suffices to complement a yeast *fpr1* mutation and restores rapamycin-sensitive cell growth [32]. Thus, all of the residues in human FKBP12 required to interact with Tor are sufficiently conserved indicating that the human FKBP12-rapamycin complex is fully proficient in binding and inhibiting the yeast Tor1 and Tor2 proteins.

The studies reported here extend this approach to define functions for the mammalian Sap protein orthologs PP6R2 and PP6R3 that appear to have been similarly conserved over the billion years of evolution separating yeast and humans from their last common ancestor. These findings suggest that PP6R1, PP6R2, and PP6R3 may play analogous mTor related signaling roles in metazoans that remain to be defined. By a similar approach recently reported by other investigators, the elements of the PI-3 kinase/PTEN/Akt cascade have been reassembled in yeast and found to provide signaling activity [33]. Thus, it may prove both technically feasible and informative to express heterologous, multi-molecular signaling circuit ensembles in the context of the yeast cell as a proxy for their functions in the much more complex milieu of the multicellular eukaryotic system.

## Materials and Methods

### Ethics Statement

N/A

### Yeast strains, plasmids, and media

All strains used in this study are derived from wild type strain MLY41 (∑1278b background) and are listed in Table 1. Construction of SCY94, JRY29, JRY40, JRY44, and JRY45 was described earlier [5]. Plasmids *pSAP185* and *pSAP155* were a kindly provided by Charles DiComo. *Kluyveromyces lactis* strain AWJ137 was obtained from Craig Bennett (Duke University). YPD-Zymocin containing plates were prepared as previously indicated [15]. All three PP6R genes containing an N-terminal Flag tag were amplified by PCR from pcDNA3-based plasmids [17] and cloned into pRS416-*ADH1* (*URA3*) [34] by homologous recombination [35].

**Table 1:** Yeast strains.

Strain	Genotype	Source
*S.cerevisiae*		
MLY41	*MAT* ***a*** * ura3-52* (∑1278 background)	Lorenz et al., 1997
SCY94	*MAT* ***a*** * ura3-52 sit4::kanMX*	Cutler et al., 2001
JRY29	*MAT* ***a*** * ura3-52 sap185::hygB sap190::kanMX*	Rohde et al., 2004
JRY40	*MAT* ***a*** * ura3-52 sap4::kanMX sap155::hygB sap185::hygB sap190::kanMX*	Rohde et al., 2004
JRY44	*MAT* ***a*** * ura3-52 sap4::kanMX sap155::hygB sap185::hygB*	Rohde et al., 2004
JRY45	*MAT* ***a*** * ura3-52 sap155::hygB sap185::hygB sap190::kanMX*	Rohde et al., 2004
*K. lactis*		
AWJ137	*MAT* ***a*** * leu2 trp1* [pGK11^+^ pGK12^+^]	Kamper et al., 1991

Unless otherwise indicated strains were grown to exponential phase in yeast extract peptone dextrose (YEPD) or synthetic complete media. Yeast synthetic media (YNB) with ammonium sulfate was supplemented with 2% glucose. SD media was supplemented with amino acids to satisfy auxotrophic requirements. Rapamycin was added to the media from a concentrated stock solution in 90% ethanol, 10% Tween20. Yeast transformations were performed using the Lithium acetate method [36].

### Amino acid sequence comparisons

Amino acid sequences of Sap155 (P43612), Sap4 (P53036), Sap185 (P40856), Sap190 (P36123), PP6R1 (Q9UPN7), PP6R2 (O75170), and PP6R3 (Q5H9R7) were obtained from the European Bioinformatics Institute (EBI, http://www.ebi.ac.uk). Sequence alignment was performed using Jalview as alignment editor [37].

### Western blotting

For immunoprecipitation of FLAG tagged PP6R proteins, whole cell extracts were prepared from exponentially growing cultures of JRY40 transformed with vector alone (pRS416-ADH) or containing each of the PP6R genes N-terminally tagged with one FLAG epitope. Cells were harvested and subjected to mechanical breakage using glass beads in lysis buffer containing 20 mM KHPO_4_ (pH 7.2), 2 mM EDTA, 2 mM EGTA, 25 mM β-glycerophosphate, 25 mM NaF, 1 mM NaVO_4_, 0.5% TritonX-100, 1 mM DTT, a mixture of proteinase inhibitors (cocktail IV-Calbiochem, La Jolla, CA), and 0.5 mM phenylmethylsulfonyl fluoride [5]. To detect the PP6R proteins, 3 mg of protein extract was immunoprecipated with 30 µl of EzviewTM Red Anti-FLAG M2 Affinity Gel (Sigma) for 2 hrs at 4°C. Immunoprecipitates were washed four times with lysis buffer. Proteins extracted from immunoprecipitated beads were electrophoresed through 4–20% Novex Tris-Glycine Gels (Invitrogen), subjected to Western blot analysis, and probed with a monoclonal antibody specific for FLAG (Sigma). The blot was stripped and reprobed with a rabbit polyclonal Sit4 specific antibody (kindly provided by Yu Jiang, University of Pittsburg). Pgk1 was detected with a monoclonal antibody (Molecular Probes).

For analysis of eIF2α, 75 µg of protein were electrophoresed through 4–20% Novex Tris-Glycine Gel (Invitrogen) and subjected to Western blot analysis. To prevent the dephosphorylation of proteins 100 mM NaF was added to the blocking milk solution. Blots were probed with an antibody specific to the phosphorylated Ser51 residue of eIF2α (BIOSOURCE International). Blots were stripped and reprobed with an anti-Sui2 antibody (a generous gift of A. Hinnebusch) to detect the eIF2α non-phosphorylated isoform.

### Flow cytometry, budding index, and cell cycle synchrony

Flow cytometry analysis were performed as previously described [38]. Cells were harvested at OD_600_ of 0.4, fixed overnight in 70% ethanol, washed with water, and incubated with 2 mg/ml RNase A (Sigma) in 50 mM Tris-HCl (pH 8.0) for 3 hrs at 37°C. Cells were washed and treated with 5 mg/ml pepsin (Sigma) in 0.45% HCl (vol/vol) for 15 min. DNA was stained with Sytox Green (Invitrogen) in 50 mM Tris-HCl (pH 7.5) and cells were briefly sonicated to separate clumped cells prior to FACS analysis. DNA content of 10,000 cells was measured with a Becton Dickinson FACSCalibur and analyzed with CellQuest software (Becton Dickinson Biosciences, San Jose, CA). The percentage of budded cells was determined as previously described [12] by counting at least 400 cells for each of three independent cultures.

For cell cycle synchronization studies, cells were elutriated as described [39]. Briefly, 1–2 liters of cells were grown at 30°C in SD-Ura to an OD_600_ of 0.8, chilled to 4°C, and sonicated to disperse clumps. The cells were loaded into an elutriator rotor (Beckman Instruments) and centrifuged at 4,000 rpm for 1 hr at 4°C. The fraction enriched in G_1_ cells was collected in bottles on ice, concentrated by centrifugation, resuspended in fresh SD-Ura media at OD_600_ 0.4, and incubated at 30°C for various times.

### Northern Blot Analysis

Yeast strains were grown overnight in SD-Ura medium to exponential phase (OD_600_ of 0.6–0.8). 100 nM rapamycin was added to the cultures and cells were harvested at various time points. RNA isolation and northern blot analysis were performed as previously described [40] and specific signals were quantified with a Typhoon 9200 variable mode imager using the Image Quantifier 5.2 software (Molecular Dynamics).
